# Bilateral pulmonary contusion, flail chest and respiratory failure on late iatrogenic diaphragmatic eventration and severe kyphoskoliosis: a case report

**DOI:** 10.1186/1757-1626-1-358

**Published:** 2008-11-28

**Authors:** Panagiotis Hountis, Sotirios Moraitis, Panagiotis Dedeilias, Nikolaos Antonopoulos, Levon Toufektzian, Mattheos Douzinas

**Affiliations:** 1 Department of Cardiothoracic Surgery, Athens Naval and Veterans Hospital, Deinokratous 70, Kolonaki, Athens, Greece; 2 Department of Cardiac Surgery, Evaggelismos General Hospital, Ipsilantou 45-47, Athens, Greece

## Abstract

**Background:**

We present a case of a 79 year old Caucasian Greek female with multiple acute and chronic diseases that in conjunction led to a critical condition.

**Case presentation:**

She had a chronic iatrogenic diaphragmatic eventration, chronic severe kyphoskoliosis and an acute thoracic trauma due to a falling. We decided to perform an operation for the diaphragmatic eventration and the flail chest on this patient in the hope of improving her respiratory capacity.

**Conclusion:**

Managing one chronic (eventration) and one acute (flail chest) thoracic disorder would restore normal mechanical properties of the thoracic cavity, although none of these diseases would be managed surgically as separate entities.

## Background

Eventration, meaning out of (E) the belly (VENTER), is a condition where all or a portion of one hemidiaphragm is permanently elevated yet retains its continuity and normal attachments to the costal margins. It remains a rare condition where surgery is seldom indicated. It can be a congenital disorder of the infantile or adult type, or a sequelae of acquired disorders with intact or abnormal phrenic nerve conduction velocity. The latter is usually a result of posttraumatic or postoperative conditions, neuromuscular or infectious disorders, malignancies of the lung or mediastinum and rarely idiopathic.[1] We present a case of a late iatrogenic diaphragmatic eventration in a previously mildly symptomatic female patient complicated by severe thoracic trauma, after a falling while riding a donkey, which lead to bilateral pulmonary contusion, flail chest of the left hemithorax and a gradual onset of respiratory failure which lead to intubation in 24 hours. This patient also had a history of severe kyphoskoliosis. We discuss the value of early operative management on weaning.

## Case presentation

**Figure 1:**
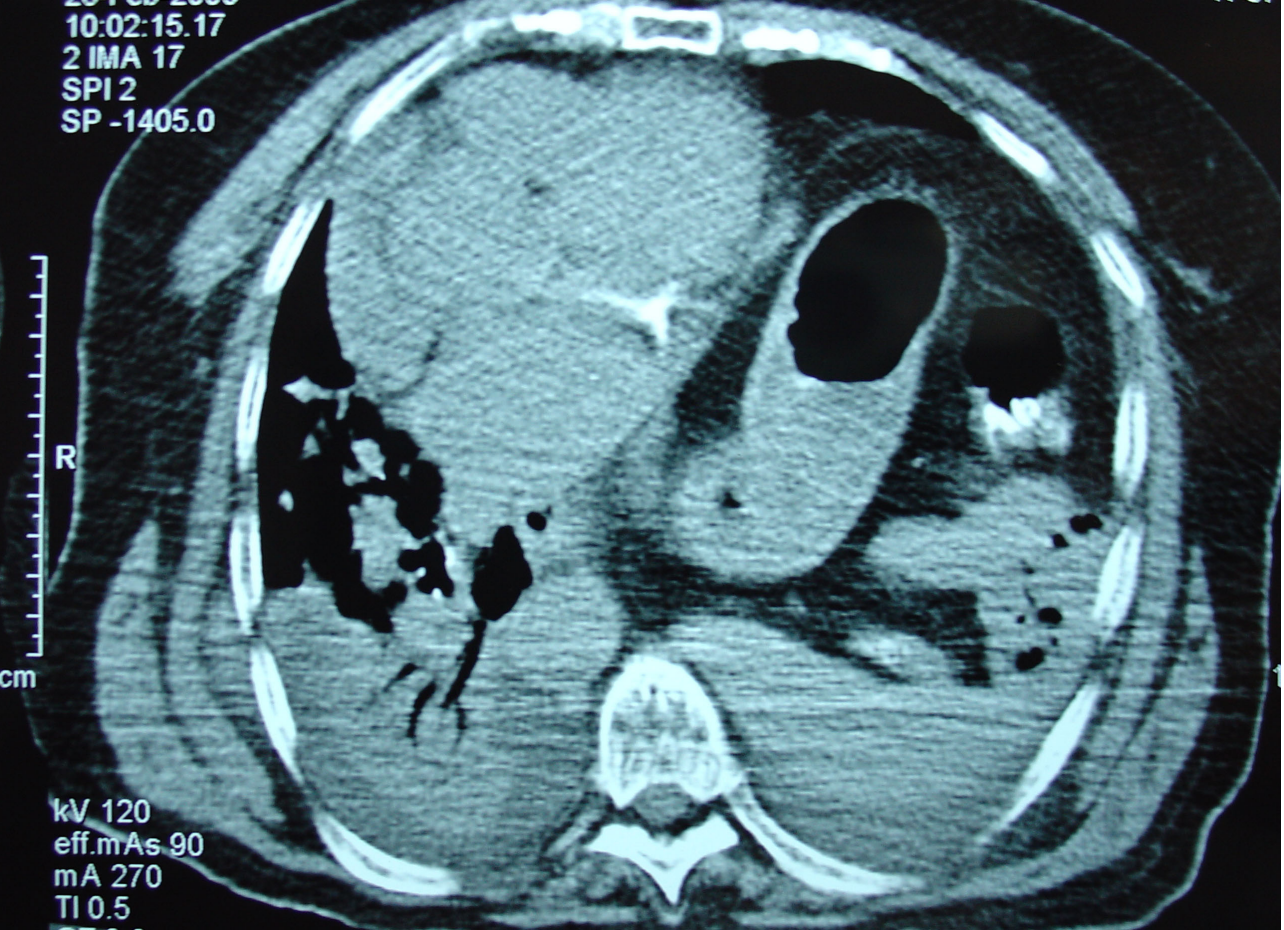
Thoracic CT scan disclosed stomach and bowel loops (chronic) as well as bilateral pulmonary contusion and small hemothoraces (acute).

A previously mildly symptomatic 79 year old Caucasian Greek female farmer, with a chronic iatrogenic diaphragmatic eventration 19 years after a Nissen fundoplication and a history of kyphoskoliosis, was transferred to the Emergency Department after a falling on the ground while riding a running donkey. She was a 158 cm height and 67 kgr weight non smoker and non drinker female. She had a history of three uncomplicated pregnancies 45–50 years ago and she didn't use any medications. The patient complained for chest pain in both hemithoraces and shortness of breath. Upon arrival she had a Glasgow Coma Scale of 15, 76 beats/min, B.P 140/70 mm/Hg, 24 breaths/min and arterial oxymetry saturation of 97%. Arterial blood gas values were: SO2 97%; p O2 88; p CO2 43; p H 7.38. Clinical examination disclosed a flailing part on the left chest wall with bruising and reduced breath sounds on auscultation as well as a resonant percussion note. Chest X-ray was diagnostic for diaphragmatic elevation, and double rib fractures of the 5th, 6th, 7th left ribs. Spiral CT scan with intravenous contrast material revealed the presence of the eventration with a question of a possible overlooked diaphragmatic rupture, and it confirmed that bowel loops and/or stomach were present in the left hemithorax.(Fig. 1) We decided to manage this patient conservatively. 18 hours later her clinical condition gradually deteriorated, and she complained of difficulty in breathing. A repeat chest x-ray and spiral CT scan offered no new diagnostic information. Arterial blood gas values were: SO2 89%; p O2 67; p CO2 55; p H 7.31. The patient was intubated and transferred to the ICU. While on respiratory support, she progressively deteriorated demanding a 90% FiO2 to manage the restrictive pattern due to flail chest and bilateral pulmonary contusion along with the previous two clinical entities. (kyphoskoliosis and diaphragmatic eventration).

**Figure 2:**
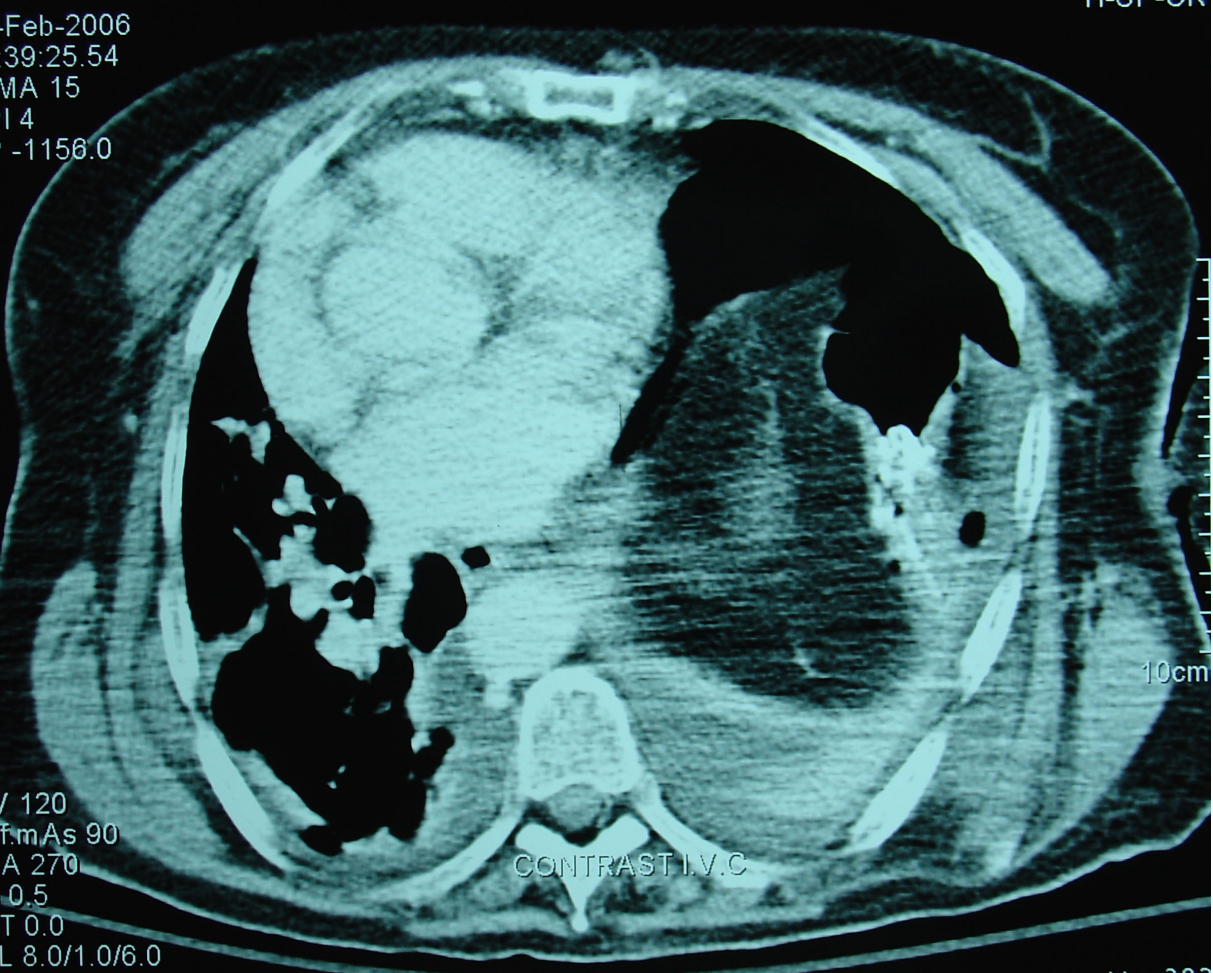
The final result on CT scan on discharge.

The decision for operative management was taken plannning to stabilize the thoracic wall and reduce the abdominal content back into the peritoneal cavity. We thought that if we allowed left lung to expand as well as explore the diaphragm for a possible misdiagnosed rupture and stabilize the flailing thoracic wall, we could offer better chance for survival. A left posterolateral thoracotomy through 5th bed rib under double lumen anesthesia was performed. At thoracotomy the diaphragm was intact but weak and floppy, and this confirmed the diagnosis of late iatrogenic eventration. We stabilized the thoracic wall with heavy figure of eight sutures, reducing the abdominal content back into the peritoneal cavity and performing a diaphragmatic plication Postoperatively the patient gradually improved. Arterial blood gases were essentially normal with gradually lowering of the inspired oxygen content and she was extubated three days later. The patient stayed in the hospital for 15 days after which she was discharged in a good general condition. CT scan on discharge disclosed a good operative result. (Fig. 2).

## Discussion

This case highlights the diversity in the surgical management of a trauma patient, especially in cases in which no apparent operation seems to be appropriate initially. We faced with a case of an old female with severe kyphoskoliosis and iatrogenic diaphragmatic eventration years after an abdominal operation. Her lifestyle and trauma history was a proof that this lady was mildly symptomatic in her daily activities. Although both of these diseases would probably pose a restrictive pattern in pulmonary capacity, the patient was in a good general condition during the first 12 hours leading us to the hypothesis that she would have a routine recovery from the flail chest and the bilateral pulmonary contusion. There was no thought for operating in the acute phase for a long standing disease or for an uncomplicated flail chest. A possible question would be whether we had a concurrent traumatic rupture on an eventrated diaphragm. In this case we were informed for the patient's history that was diagnosed with eventration shortly after the Nissen fundoplication. We performed an upright chest radioscopy that was diagnostic for the unbalanced but otherwise intact movements of the left hemidiaphragm.

In any other case, without such a history, the most prominent diagnosis would be traumatic diaphragmatic rupture which has an incidence of 2–5% in closed thoracoabdominal injuries. Early diagnosis and repair of the defect are important in avoiding the potentially devastating sequelae of herniation and strangulation of abdominal viscerae. In general early repair should be via the abdominal route in order to deal with any associated intabdominal visceral injuries whereas late repair is via the thoracic route, as adhesions to thoracic structures can be more easily dealt with.[2] When our patient showed rapid clinical worsening we decided to intubate her in order to facilitate respiratory recovery from bilateral pulmonary contusion and flail chest. Respiratory failure seemed inevitable and it was prominent that this patient would not be able to be managed by intubation alone. On the other hand a trauma patient of this age would not be able to survive from the possible complications of a long lasting ICU stay, especially infections.

Management of diaphragmatic eventration varies greatly depending on whether the diagnosis is made in newborns or infants, or in adults. Most cases of eventration occurring in adult life should be treated conservatively unless severe dyspnea interfering with normal activities, orthopnea, or gastrointestinal symptoms are clearly related to the high position of the diaphragm.[3] Having in mind that she had an essential normal life activity despite of the eventration and kyphoskoliosis, we thought that early surgical management could be beneficial for this patient for three reasons.

Firstly, by reducing, at this moment, abdominal content back would help left lung to expand and increase respiratory capacity. Secondly we had an opportunity to fix flail chest, that serves the same purpose: Early extubation and return to a routine recovery program. Finally, although we had a radioscopy diagnosis that diaphragm was intact it would be a possible overlooked traumatic diaphragmatic rupture.

## Conclusion

Although this patient suffered from three different clinical entities, not usually treated by surgical route, we decided to perform this operation in order to improve her respiratory status and thus lessen the need for mechanical ventilation. This would help avoiding the devastating complications associated with prolonged ICU stay. On the other hand this could help us institute a definite diagnosis. This case focuses on the different access that can be performed on a given patient, according to his medical history, rapidity of the events, clinical condition as well as radiology exams. We would like to emphasize on the concept that 'surgical disease is either treated by an operation or it may be treated by an operation during its course'.

## Abbreviations

B.P: Blood pressure; SO2: Blood Oxygen saturation; p O2: partial oxygen pressure in the blood gases; p CO2: partial carbon dioxide pressure in the blood gases; p H: negative logarithm of the Hydrogen ion concentration; X-ray: classic Radiography; ICU: Intensive care unit; FiO2: Percentage of inspired oxygen; CT: Computed Tomography.

## Consent

"Written informed consent was obtained from the patients for publication of this case report. A copy of the written consent is available for review by the Editor-in-Chief of this journal."

## Competing interests

The authors declare that they have no competing interests.

## Authors' contributions

PH was a major contributor in writing the manuscript.  SM, PD, NA and LT analyzed and interpreted the patient data. MD took care and operated the patient.

All authors have read and approved the final manuscript.
